# RFamide Peptides: Structure, Function, Mechanisms and Pharmaceutical Potential

**DOI:** 10.3390/ph4091248

**Published:** 2011-09-21

**Authors:** Maria Findeisen, Daniel Rathmann, Annette G. Beck-Sickinger

**Affiliations:** Institute of Biochemistry, Leipzig University, Brüderstraße 34, 04103 Leipzig, Germany; E-Mails: mfind@uni-leipzig.de (M.F.); rathmann@uni-leipzig.de (D.R.)

**Keywords:** RFamide, neuropeptide FF, kisspeptin, prolactin-releasing peptide, QRFP

## Abstract

Different neuropeptides, all containing a common carboxy-terminal RFamide sequence, have been characterized as ligands of the RFamide peptide receptor family. Currently, five subgroups have been characterized with respect to their N-terminal sequence and hence cover a wide pattern of biological functions, like important neuroendocrine, behavioral, sensory and automatic functions. The RFamide peptide receptor family represents a multiligand/multireceptor system, as many ligands are recognized by several GPCR subtypes within one family. Multireceptor systems are often susceptible to cross-reactions, as their numerous ligands are frequently closely related. In this review we focus on recent results in the field of structure-activity studies as well as mutational exploration of crucial positions within this GPCR system. The review summarizes the reported peptide analogs and recently developed small molecule ligands (agonists and antagonists) to highlight the current understanding of the pharmacophoric elements, required for affinity and activity at the receptor family. Furthermore, we address the biological functions of the ligands and give an overview on their involvement in physiological processes. We provide insights in the knowledge for the design of highly selective ligands for single receptor subtypes to minimize cross-talk and to eliminate effects from interactions within the GPCR system. This will support the drug development of members of the RFamide family.

## 1. Introduction

Neuropeptides characterized by a common carboxy-terminal arginine (R) and an amidated phenylalanine (F) motif (designated RFamide peptides) were originally discovered in invertebrates [1]. In mammals it is now known that there exist at least five genes, encoding the family members and the five G-protein coupled receptors through which RFamide peptides act. All these peptides show a great diversity, regarding their N-termini and hence a wide pattern of biological activity.

The initially identified tetrapeptide FMRFamide was isolated from the ganglia of the clam as a cardioexcitatory peptide [1]. Significant improvements in the molecular characterization of the RFamide peptide system have been made since then. Nowadays the rising number of RFamide peptides found in mammals can be subdivided into five groups: the neuropeptide FF (NPFF) group, the gonadotropin-inhibitory (GnIH) group, the 26RFa group, the kisspeptin/metastin group and the prolactin-releasing peptide (PrRP) group. By applying a reverse pharmacological method and by searching of DNA sequence databases several other structurally similar peptides have also been identified (Table 1). The first reported vertebrate member was LPLRFamide [2], and the first mammalian RFamide peptides were neuropeptide FF and neuropeptide AF [3]. Both were purified from bovine brain extracts [3], showed several biological activities *in vivo* in mammals [4] and are encoded by a single gene [5]. GnIH homologs were isolated from extracts of the human hypothalamus by immunoaffinity purification, and furthermore identified as human RFamide-related peptide 1 (RFRP-1) and human RFRP-3 by mass spectrometry [6]. Both, 26RFa and the longer form of 26RFa, termed 43RFa (QRFP), have been isolated from the human hypothalamus and spinal cord [7].

PrRP was identified in 1998 by a reverse pharmacology approach [8]. Up to now two equipotent isoforms of different N-terminal length are known, PrRP31 and PrRP20 [9]. The naturally occurring kiss RFamide peptides, consisting of 54-, 14-, or 13-amino acids were isolated from human placenta [10], whereas the highly potent kisspeptin-10 was synthesized [14]. Due to the prediction that all three endogenous peptides derive from KiSS-1 [11], a metastasis suppressor gene for melanoma cells, they were designated as kisspeptins. It should be noted that Hori *et al.* [12] named the same peptide metastin, which derives from its initially discovered function to suppress metastasis, but for clarity kisspeptin is used preferentially in this review.

## 2. RFamide Peptide Receptor Family

Several orphan receptors turned out to respond to different members of the RFamide peptide family (Table 2). The PrRP receptor was the first putative RFamide receptor that was identified and initially described as GPR10 [8]. In 2000, the putative receptors for NPFF and NPAF were identified and named NPFF1/GPR147 and NPFF2/GPR74 [13,16,17]. Independently, two groups could show that both, 26RFa and 43RFa, act as natural ligands of the orphan receptor GPR103 [18,19]. The kisspeptin receptor or KiSS1-derived peptide receptor was formerly discovered as orphan G-proteincoupled receptor GPR54 [20,22]. For the explanations that follow we refer to the protein names of the corresponding receptors (Table 2).

So far there are only few mutagenesis data available. We decide to focus on one residue on the top of transmembrane helix (TMH) 6, which is known to be present and/or important for receptor activation in several related receptor families (e.g. the NPY receptor family). Because all ligands share an amidated dipeptide motif at their C-termini with the positively charged arginine side chain, an acidic residue has been proposed to act as a counterpart. Therefore, first investigations were made on an acidic residue 6.59 on the top of TMH 6, according to the nomenclature of Ballesteros and Weinstein [23].

### 2.1. Neuropeptide FF Receptor 1 (GPR147)

It was recently shown by immunohistochemistry that cells expressing the human NPFF_1_R are localized in the human hypothalamus and surrounding areas [24]. It is now clear, that most human forebrain nuclei contain such cells, supporting the idea that RFamide peptides play a role in central coordination of neuroendocrine and autonomic responses in humans. The NPFF_1_R commonly activates the G_i/o_ signal transduction pathway [13,16]. The human receptor was isolated from a human spinal cord cDNA library and analysis by sequencing led to a coding sequence of 1290 bp, and a predicted protein with a length of 430 amino acids [16]. Comparing the primary sequence of human NPFF_1_R with other known GPCRs showed, that NPFF_1_R is most similar to human orexin1 (37% identity), human orexin2 (35%), human neuropeptide Y receptor subtype 2 (NPY_2_R) (34%), human chole-cystokinin A (CCKA) (34%), human NPY_1_R (32%), mouse GIR (32%), human prolactin-releasing hormone receptor (32%), and human NPY_4_R (31%) [16]. RFRP-1 and RFRP-3 efficiently inhibit production of forskolin-induced cAMP accumulation in CHO cells, expressing the human NPFF_1_ receptor [13]. Furthermore, pharmacological characterization of hNPFF_1_-transfected SH-SY5Y cells was performed by measuring the intracellular cAMP content [25]. By using a [^35^S]GTPγS binding assay, the functional activities of RFamide peptides were investigated in CHO cells stably expressing the NPFF_1_ receptor [26].

To the best of our knowledge, there is just one receptor mutant published so far. In studies regarding the related NPY receptor family, Merten *et al.* could clearly demonstrate that the acidic residue 6.59 on the top of TMH 6 plays an important role in ligand binding [27]. Thus, Findeisen *et al.* presumed a strong ionic interaction to be involved in ligand recognition and receptor activation at position 6.59. When the derived Asp^6.59^ mutants were investigated [28], the introduction of an elongated charge conserving glutamate revealed a minor loss in receptor activation, whereas the replacement of Asp with Ala or Asn led to a significant loss in affinity and receptor activation. The need for a negatively charged side chain at position 6.59 is underlined by the introduction of Arg, resulting in strongly decreased affinities and a not fully activatable hNPFF_1_R mutant. Another part of the binding pocket is proposed to be built up by residues located in the upper sections of the TMHs. Further investigations revealed evidence that other hydrophobic residues are not involved in a direct interaction. By mutating putative residues of impact to alanine, we merely found a minor loss of receptor activation for Phe^5.43^, Phe^7.37^, Phe^7.44^ (unpublished data).

### 2.2. Neuropeptide FF Receptor 2 (GPR74)

By autoradiography and by the use of a radiolabelled NPFF analog, [^125^I](1DMe)Y8Famide, it could be shown that NPFF receptors are localized in the central nervous system [29,30]. The NPFF_2_R mostly activates the G_i/o_ signal transduction pathway [16,17]. The human full-length receptor was amplified from spinal cord cDNA and further analysis by sequencing led to a coding sequence of 1260 bp, and a predicted protein with a length of 420 amino acids [16]. Both NPFF receptors share 50% amino acid identity. Pharmacological characterization of human NPFF_2_ receptor was performed by measuring the cAMP levels in CHO cells as well as in HEK293 cells transfected with hNPFF_2_R [17]. Furthermore, the anti-opioid effects on Ca^2+^ mobilization were investigated by measuring Ca^2+^ contents in SH-SY5Y cells transfected with the human NPFF_2_R [31]. Additionally, the [^35^S]GTPγS binding was studied in CHO cells stably expressing the human NPFF_2_R [26].

To compare the receptor subtype specific activation the same acidic residue on the top of TMH 6 was investigated by Findeisen *et al.* [28]. The introduction of glutamate at position 6.59 revealed an equipotent minor loss in NPFF_2_R activation as for NPFF_1_R. Regarding NPFF receptor subtype selectivity the modifications of Asp to Asn and Ala was better tolerated at the hNPFF_1_R, compared to the hNPFF_2_R. This clearly indicates that the hNPFF_2_R is more sensitive at this receptor site. The replacement of Asp^6.59^ with Arg resulted in a not fully activatable hNPFF_2_R variant, which demonstrates the necessity of a negatively charged amino acid. Further investigations regarding hydrophobic residues in the upper transmembrane regions revealed no involvement in receptor activation. By mutating residues of interest to alanine we could see no loss of receptor activation at position Phe^5.43^ or Phe^7.40^ (unpublished data).

### 2.3. Pyroglutamylated RFamide Peptide Receptor (GPR103)

The QRFP receptor is an orphan G-protein coupled receptor and was originally cloned from a human brain cDNA library. It has been detected widely throughout the brain, heart, kidney, retina and testis [19]. GPR103 encoded a 455 amino acid protein and phylogenetic analysis showed that the orphan receptor shares identities with peptide-binding receptors, including NPFF_2_R, NPY_2_R and galanin GalR1 receptors (34–38% in the TM regions). The QRFPR commonly activates the G_q_/G_i_ signal transduction pathway. With regards to the conserved acidic residue at position 6.59, a glutamate is present instead of an aspartate. Nevertheless, by the replacement with alanine, we could see the same loss (∼60-fold) in activity as for the NPFF_1_R (unpublished data). Replacement of a hydrophobic residue (Phe^5.41^) by alanine revealed no involvement in receptor activation since no decreased EC_50_-value was detected (unpublished data).

### 2.4. Kisspeptin/Metastin Receptor (GPR54)

The kisspeptin receptor was originally cloned from rat brain, sharing 45% identity with the galanine receptors [22]. In humans, reverse transcriptase polymerase chain reaction has revealed, that GPR54 was highly expressed in placenta, pituitary, pancreas, and spinal cord, suggesting an important role in the regulation of endocrine function [10,15]. Furthermore, by immunohistochemistry kisspeptin receptor protein expression was detected in the cerebral cortex, thalamus, pons-medulla and cerebellum [20]. Together with the use of radioligand binding Mead *et al.* found expression in aorta, coronary artery and umbilical vein [32]. Functional assays were performed in CHO cells transfected with the kisspeptin receptor [12] and *in vitro* pharmacology was carried out by using human isolated vessels [32]. Up to now it is known that the activated kisspeptin receptor acts via G_q_/G_11_ signal transduction pathways, leading to the stimulation of phospholipase C and consequently to calcium mobilization. Additionally, activation of the kisspeptin receptor has been shown to stimulate arachidonic acid release [10].

While the acidic residue 6.59 is conserved throughout the whole RFamide peptide receptor family, in the human kisspeptin receptor an alanine is present at that position. To our knowledge, no mutational studies have been performed so far. It should be noted that by using polymorphism scanning and typing of KiSS-1/GPR54 gene, Chu *et al.* recently unveiled a couple of harmful polymorphisms [33]. This includes the mutation of residue Phe in position 272 to Ser in the GPR54 gene, a loss-of-function mutant, which is associated with familial normosmic IHH (idiopathic hypogonadotropic hypogonadism) [34]. Underdeveloped external genitalia and impuberism point to the major role of GPR54 in the activation of the gonadotropic axis from intrauterine life to adulthood [34].

### 2.5. Prolactin-Releasing Peptide Receptor (GPR10)

The prolactin-releasing peptide receptor was originally isolated from rat hypothalamus [35] but has been detected widely throughout the human and rat brain [36]. After binding of the endogenous ligand the activated receptor most commonly activates the G_q_ signal transduction pathway [9].

The only manuscript concerning mutagenesis studies which is published so far reports that the PrRP receptor has a PDZ domain binding motif in its C-terminal tail (-SVVI) [37]. In a mutational study with the prolactin releasing peptide receptor we were able to show the involvement of the aspartic residue at position 6.59 (unpublished data). By replacement with alanine, a 22-fold loss of receptor activity upon stimulation with PrRP was observed. As mentioned above a second part of the binding pocket is proposed to be built up by residues, located in the upper sections of the transmembrane helices. Therefore, in a first study we investigated the involvement of hydrophobic residues. We could not detect a decreased EC_50_-value, when Phe^2.72^, Trp^5.40^, Phe^7.31^ or Trp^7.40^ was mutated to alanine upon stimulation, which revealed no involvement in ligand binding and receptor activation (unpublished data).

## 3. Physiological Effects of RFamide Peptides

### 3.1. Neuropeptide FF

NPFF was initially identified to attenuate morphine induced antinociception and to evoke an increased sensitivity to pain, called hyperalgesia [3,38]. Since then, the range of activities has been expanded and is associated with a plethora of physiological and behavioral phenomena. In rats, NPFF was shown to participate in the modulation of the cardiovascular system, basically inducing increased blood pressure, bradycardia or inhibition of the cardiac component of the baroreceptor reflex [39-45]. Further studies revealed an anorectic impact of NPFF in rats [46,47] and in chicken [48] but also NPAF [49] and NPSF [50] showed reduced food consumption in chicken. This effect on food consumption is generally described for almost all RFamide peptides in several vertebrates [51,52]. Furthermore, NPFF is known to be involved in water balance [47,53,54] and with NPSF and NPAF affects adipogenesis [55]. Moreover recent investigations in rodents describe NPFF to be involved in locomotion and reward [56-59]. Central injections of NPFF in mice evoked hypothermic effects [60-62] and could be antagonized by BIBP3226 as well as by RF9 [63,64]. No intrinsic affinity at any of the opioid receptors subtypes was observed for NPFF [4,65,66], nevertheless NPFF has been recognized as an effective modulator of opioid functions and pain. Additionally, NPFF was found to take part in the development of opiate tolerance and dependence [67,68], albeit the observed anti-/pro-opioid effects of NPFF are dependent on the site of administration [38,69].

### 3.2. PrRP

PrRP has been initially identified as a prolactin-releasing factor [8] but follow-up studies rather suggest that the major effects of this neuropeptide are different. In rodents, PrRP is involved in the control of body weight homeostasis inducing anorexia but it has, in contrast to NPFF, no impact on locomotion, exploratory time, grooming and resting time [70,71]. Further studies revealed evidence for a role of PrRP as a regulator for stress response [72-74], nociception [74], and the cardiovascular system, *i.e.* arterial blood pressure [75]. PrRP effects were additionally described to be mediated by the NPFF receptor system *in vivo* and *in vitro* [76] but also via the corticotropin releasing hormone system [77], which is involved in the mediation of cardiovascular [78] or anorectic effects [79].

### 3.3. Kisspeptin/Metastin

Due to its implication as a suppressing factor for metastasis, the processed 54 amino acid peptide kisspeptin was originally named metastin [10-12,15]. The role of the kisspeptin/metastin encoding *Kiss1* gene in cancer metastasis has been recently reviewed [80]. The common perception of metastin/kisspeptins as pivotal regulator for reproduction [81-84] was the inception for a plethora of studies in this field. Either central or peripheral administration of the peptides kisspeptin-54 and/or kisspeptin-10 were identified to stimulate the gonadotropin-releasing hormone (GnRH), luteinizing hormone (LH), follicle-stimulating hormone (FSH), and testosterone secretion [81-84]. Furthermore, the importance of the kisspeptin receptor GPR54 was elucidated as some cases of hypogonadotopic hypogonadism were associated with mutations of the GPR54 gene [85-87]. Mice, lacking the gene of the receptor fail to undergo sexual maturation [86]. In contrast to the other RFamide peptides, kisspeptin/GPR54 is not directly involved in body weight control and food consumption [86,88]. It was only described to reduce food consumption by increasing the meal intervals in mice [89] or the reduced hypothalamic expression of kisspeptin itself was observed for leptindeficiencyanddiet-induced obesity in mice [90]. The multiple physiological functions of the kisspeptin/kisspeptin receptor signaling as a nodal point in the neuroendorinical regulation of puberty and reproduction [91] have been recently reviewed and are not focus of the present review [92-95].

### 3.4. GnIH

The name of the gonadotrophin-inhibitory hormone already describes the initially discovered function for the RFamide related peptide, which was discovered in 2001 in quail brains [96]. Further exploration identified the mammalian orthologs of the avian GnIH, called RFamide related peptide-1, -2 or -3 (RFRP-1, -2, and -3) and their functional roles. RFRP-3 application in rodents confirmed the inhibitory effects on GnRH neurons [97] and further investigations suggest an inhibitory modulation of GnRH stimulated LH secretion at the pituitary [98,99]. But RFRP-3 has also a strong influence on feeding and sexual behavior in rats [100]. In ovine, RFRP-3 was shown to project to cells being involved in regulation of energy balance and reproduction [101]. For clarification, it has to be emphasized that the human RFRP-3 shares the same sequence with the ovine RFRP-3 and more importantly, it is also designated as NPVF in humans. Previous investigations reported NPVF as NPFF_1_R selective agonist, which attenuate morphine-induced antinociception more effective than NPFF. Furthermore, NPVF is suggested to be important for the endogenous anti-opioid mechanism [25,102,103]. The hyperthermic effects of NPVF resemble those of NPFF [62,63]. In chicken, NVPF regulates appetite in a short term manner and its effects are related with hypothalamic and behavior changes [104]. The NPVF-induced satiety was shown to be mediated through μ and κ but not δ subtypes of opioid receptors in chicken [105].

### 3.5. 43RFa (QRFP)/26RFa

The latest member of the RFamide peptides, 26RFa or 43RFa (QRFP) demonstrates a variety of different physiological processes to be involved in, such as regulation of the cardiovascular system by elevating the arterial blood pressure and the heart rate in rodents [106,107]. Administration of either 26RFa or QRFP plays an important role in energy homeostasis by regulating the appetite and energy expenditure, finally resulting in obesity with hyperphagia [14,107-110]. Additionally, both ligands possess the ability to activate the gonadotropic axis [111,112] and can increase locomotor activity [107,109]. Recent data present 26RFa able to produce an analgesic effect [113] and to modulate the nociceptive transmission, finally evoking anti-allodynic effects [114]. Furthermore, 26RFa is suggested to promote bone formation [115]. This is suggested by the identification of the 26RFa gene as a potential osteoporosis gene [116].

### 4. Structure-Activity and Structure-Affinity Studies

A common approach to identify the most important segment of a ligand is to determine the minimal sequence, which is required to activate the receptor. To note, the first described structure-activity study of an RFamide peptide on molluscan muscle revealed that FMRF-OH retained 1/100th of the activity of FMRFamide, while MRFamide had 1/1000th [1]. Thus, the amidation as well as a certain peptide length had to be considered for future SAR studies at vertebrates. Indeed, the amidated C-terminal fragment of the ligand family is essential for all receptors within this family. Structure-affinity/activity relationship studies can be used to characterize the interaction between ligand and receptor. Essential segments of the peptide and essential segments of the receptor for the interaction can be identified and distinguished from non-essential residues. Furthermore, the investigation of the three-dimensional structure of biologically active peptides is important for the clarification of structure-activity relationships and thus for the development of potent agonists and antagonists [117].

### 4.1. SAR of NPFF Analogs

Many modifications of the NPFF sequence on the N-terminus and the C-terminus have been performed [118,119]. The affinities of NPFF-related peptides inhibiting [^125^I]1DME or [^125^I][Tyr^1^] NPFF specific binding in the dorsal horn of rat spinal cord are summarized in Table 3. First investigations clearly showed that neither an acetylated N-terminus nor deletion of the two N-terminal residues (not shown) significantly modified peptide affinity [118,119]. In contrast, NPFF(4-8) and NPFF(5-8) showed a 100-fold reduced affinity compared to NPFF. Further on, the carboxy-terminal tripeptide itself (Gln-Arg-Phe-NH_2_) had only a weak affinity (K_i_ of 300 ± 45 nM) [118,119]. The introduction of the *D*-enantiomer at position 5 reduced the affinity 100-fold. Accordingly, the most important part is the RFamide motif. Substitutions at position 7 or 8 are not well tolerated and reduced the affinity dramatically. Only substitution to *D*-Arg in position 7 or Tyr in position 8 showed a moderate loss of affinity about 100-fold compared to NPFF [118,119]. Double substitution of Arg^7^ and Phe^8^ by the corresponding enantiomer (*D*-Arg^7^ and *D*-Phe^8^) significantly reduced the affinity. The amidated C-terminus is as important as the last two residues. The free acid containing peptide does not show any significant potency compared to the amidated form [118,119].

The classic strategy of amino acid substitution and deletion could clarify the structural requirements for binding to the NPFF receptors. Since then, signal transduction assays were performed in combination with binding experiments on recombinantly expressed NPFF receptor subtypes. The data in Table 4 provide information about the receptor subtype specificity, with regards to the pro-NPFF_A_ and pro-NPFF_B_ derived peptides. These two precursors generate different RFamide peptides. It has been suggested that peptides derived from pro-NPFF_A_ display high affinity/activity at the NPFF_2_R and, conversely, peptides from pro-NPFF_B_ precursor show slight preference for the NPFF_1_R [102]. Data are similar between the different functional assays and confirm that RFRP-3 (NPVF) and RFRP-1 (NPSF) are more active at the NPFF_1_R than NPFF and NPAF. In accordance with other data, the opposite behavior was found at the NPFF_2_R for NPFF, NPAF and RFRP-3, RFRP-1. Furthermore, shortening the NPFF sequence clearly underlines that these modifications are better tolerated at the NPFF_2_R. And *vice versa* modifications in the RFRP-3 and RFRP-1 sequence show greater effects at NPFF_1_R [28,120,121]. In contrast, by ending up in the tetrapeptide PQRF-NH_2_ the same behavior is displayed for both receptors [28,120,121]. This is not surprising because of the equal carboxy-terminal four amino acids.

As previously reported [119,120], substitutions of the C-terminal Arg and Phe residues result in a significant loss in receptor response. By the use of a systematic approach, we characterized the role of the C-terminal dipeptide with respect to agonistic properties. The synthesized [Xaa^7^]NPFF and [Xaa^8^]NPFF analogs were used to investigate signal transduction properties in COS-7 cells, transiently expressing human NPFF receptor subtypes [28]. Data are summarized in Table 5.

Substitution of Arg with Ala resulted in a peptide that maintained weak activity, but only at high concentrations (> 1 μM) for NPFF_1/2_R. Findeisen *et al.* [28] were able to further explore the crucial role of Arg^7^ by modifying this position and show that all alterations resulted in identical behavior at the NPFF_1_R and NPFF_2_R: the charge-conserving Lys residue displays the same behavior at both receptors, inducing a ∼100-fold decrease in potency for IP accumulation at NPFFRs. Furthermore, to assess the importance of the cationic guanidinium group within the Arg^7^ side chain of the NPFF sequence, terminal NH-groups were monomethylated (MMA) or asymmetric dimethylated (ADMA), resulting in analogs that show an equal activity profile at both NPFFRs as well. No significant change in efficacy was observed for either receptor subtype relative to stimulation with parent NPFF. Interestingly, by decreasing the Arg^7^ side chain by either one or two CH_2_ groups [replacement by α-amino-4-guanidino-butyric acid (Agb) or α-amino-3-guanidinopropionic acid (Agp)], the resulting analogs exhibit different activity profiles for both receptors. Low potency was observed for both receptors, but the efficacy was different: the [Agb^7^]NPFF analog was only able to activate the NPFF_1_R up to 31 ± 2% of the wild-type efficacy in contrast to the maximum signal of 96 ± 13% observed for NPFF_2_R. Other hydrophilic amino acids, such as Asp and Cit resulted in dramatically decreased receptor activation. Findeisen *et al.* postulated that the length of the side chain is important, and that various molecular dynamics are responsible for formation of the agonist-receptor complex in the NPFF_1_R as compared with the NPFF_2_R [28]. The recently discovered selective NPFF_2_R agonist with a guanidinium group positioned very close to the core structure supports this hypothesis (compounds 1, 3 and 9; Table 10) [123]. These results are in agreement with the requirement of a long, charged side chain with hydrogen bonding potential at this position, suggesting that the specific role of the Arg is critical for best activation of both NPFF receptors.

To study the critical role of the C-terminal phenylalanine, a series of [Phe^8^]NPFF analogs with aromatic and aliphatic amino acids, as well as modifications of the Phe side chain, was investigated by comparing the activity profiles (Table 5). The substitution with alanine led to nearly complete loss of receptor activity for both subtypes, which is in agreement with previous binding studies using the dorsal horn of the rat spinal cord [119]. Interestingly, the activity was essentially equipotent at both NPFF receptor subtypes after replacement with a cyclohexyl group at position 8 of NPFF and resulted in an approximate five-fold decreased potency. The presence of a hydroxy group in the Tyr side chain resulted in an agonistic peptide with ∼24-fold loss in potency for both NPFFRs.

In contrast, substitution of Phe^8^ with Trp or His did not generate potent peptides for NPFF_1_R. Both peptides exhibit agonistic activity, but their efficacy was drastically reduced. However, the C-terminal Phe^8^ was able to be replaced by Trp or His with only a minor loss in potency at the NPFF_2_R relative to the NPFF_1_R. Still some agonism was observed by the use of methylated Phe analogs. Introduction of the corresponding enantiomer caused a significant loss in potency for both receptor subtypes. Elongation of the side chain by replacement with homophenylalanine (Hph) resulted in a peptide that displayed a significant loss in activity at NPFF_1_R (147-fold) and at NPFF_2_R (444-fold). Remarkably, shortening the Phe side chain to a phenylglycine (Phg) resulted in equipotency for both NPFFRs, although the peptide has decreased efficacy at the NPFF_1_R. With respect to the overall peptide structure, we observed that configuration of the Phe^8^ side chain was of similar importance for both subtypes. Additionally, both NPFF receptors were able to be activated by analogs in which the aromatic group is larger than Phe itself (pMePhe). However, only NPFF_2_R retained the full maximum signal when the aromatic ring linker is CH_2_-CH_2_ (Hph) or absent (Phg), while repositioning of the phenyl group in relation to the peptide backbone was critical for full NPFF_1_R activation. The results propose that the agonist-receptor complexes for NPFF_1_R are more susceptible to structural modifications (Table 5).

### 4.2. SAR of RFamide-Related Peptide 26RFa

Chartrel *et al.* have isolated a 26 amino acid RFamide peptide from frog brain (hence 26RFa) [14]. Later, an N-terminally extended form of 26RFa, termed 43RFa or QRFP, was identified and purified from rat brain extract [107]. Both peptides, 26RFa and 43RFa have been isolated from the human spinal cord and hypothalamus [7]. The amino acid sequence of the carboxy-terminal heptapeptide of 26RFa has been fully conserved from fish to mammals [124] and is probably processed *in vivo* by prohormone convertases. In previous studies it has been demonstrated, that in mammals the amidation of the C-terminus is essential for biological activity of all RFamide-related peptides [119,125]. Previous data showed that the desamidated 26RFa has a 400-fold loss in affinity for GPR103 compared to the amidated analog [18].

By measuring the potency to induce [Ca^2+^]_i_ mobilization in Gα_16_-hGPR103-transfected CHO cells, structure-activity studies of a series of analogs revealed that the 26RFa is equally potent as the N-terminally elongated 43RFa, whereas the conserved heptapeptide 26RFa(20-26) was substantially less potent (75-fold), but as efficacious as h26RFa to increase [Ca^2+^]_i_ [126]. Further on, by using an Ala-scan the authors could show that the last three carboxy-terminal residues Phe-Arg-Phe are involved in the activation of human GPR103. The conserved heptapeptide can be optimized at position 23, by replacing a serine with a norvaline residue to gain a slightly more potent analog compared to the 26RFa(20-26) (Table 6). They investigated the contribution of the C-terminal carboxamide to the biological activity by using a series of mono- and disubstituted amide 26RFa(20-26) analogs. With these experiments, they could demonstrate that the C-terminal primary amide is involved in receptor binding via a hydrogen bond [126]. The gradual decline in the biological activity observed with the N-terminally truncated analogs has to be correlated with the affinity of these fragments for GPR103 [18]. Up to now this is the only activity study, which is published so far.

Regarding to the peptide structure, Thuau *et al.* described the NMR conformation of 26RFa in water and methanol as well as CD spectroscopic studies. Experiments in methanol suggest that the Pro^4^-Arg^17^ region of 26RFa adopts a well-defined α-helical structure [127]. Further on, the helix is flanked by two unstructured regions, a three amino acids short N-terminally one (Thr^1^-Gly^3^) and a longer segment consisting of the C-terminally conserved moiety (Lys^18^-Phe^26^). Both, CD- and NMR-studies, reveal a helix with amphiphatic character consisting of a hydrophobic (residues Leu^5^, Leu^8^, Ala^9^ and Leu^12^) and a hydrophilic face (residues Asn^7^, Glu^10^, Glu^11^, Asn^13^ and Arg^17^) [127].

### 4.3. SAR of PrRP

SAR for the PrRPR and its equipotent agonistic ligands PrRP20 and PrRP31 are quite clear. Early findings revealed that the analog with the C-terminal acid of PRP31 is inactive [8,125]. [^125^I]-PrRP20 is described as a high affinity ligand, whereas other peptides like NPY, NPFF, RFRP-1 or RFRP-3 show affinities > 30 μM [9,128]. In a down-sizing attempt, Roland *et al.* identified the heptapeptide PrRP25-31 to be the minimal active agonist fragment and confirmed the importance of the C-terminal amidation for the biological activity of PrRP. Using an Ala-scan they figured out that the three Arg residues at positions 23, 26 and 30 are of importance [129]. Danho *et al.* found that Ac-PrRP-(26-31)-hexapeptide was the smallest agonist sequence, and concluded that the critical amino acids were Arg^26^, Pro^27^, Val^28^, Arg^30^ and Phe^31^ [130]. The findings of an L-shaped peptide structure with a sloppy N-terminal region and a hydrophobic cluster consisting of Pro^27^; Val^28^ and Phe^31^ were confirmed by recent CD- and NMR-studies revealing an amphiphilic helix of the carboxy-terminal region [130,131]. The first comprehensive study on structure-activity relationships at a plethora of PrRP(19-31) analogs was performed by Boyle *et al.* [125] (Table 7). The data confirmed the role of the functionally important residues that are located within the carboxy-terminal heptapeptide segment Ile^25^-Arg^26^-Pro^27^-Val^28^-Gly^29^-Arg^30^-Phe^31^-NH_2_, especially of residues Arg^26^, Arg^30^, Phe^31^ as well as the C-terminal amide function. Repositioning of the aromatic ring at Phe^31^ was not tolerated and substitution of aliphatic or polar residues resulted in little functional activity. In contrast, substitutions of the aromatic ring in correct distance to the backbone were well tolerated.

No functional activity was observed for any modification made at position Arg^30^, emphasizing its crucial role, whereas Arg^26^ is also relevant but has reduced impact [125]. Gly^29^ is very important for good functional activity and even slight changes are poorly tolerated. Pro^27^ is probably required for its turn-promoting property as modifications resulted in less affinity. Val^28^ could be replaced by Phg with full retention of functional and binding activity, indicating that it is supplying a reasonably sized hydrophobic side chain in the L-configuration and exhibiting branching close to the peptide backbone. Ile^25^ also accepts substitution by Phg but it seems to be a less important position as Ala in position 25 also retained considerable functional activity. The data obtained from a minor number of analogs in positions 21 to 23 confirm that these residues are less important. Backbone methylation from positions 26 to 31 resulted in reduced or none functional activity [125].

Recently, the investigations at position Phe^31^ have been intensified and confirmed the important function of Phe^31^ PrRP analogs *in vivo* and *in vitro*, with respect to binding, activity, and food intake in fastened mice after central administration [132]. In detail, analogs with deleted Phe or containing Phe derivatives with bulky side chain or halogenated aromatic ring were tested and revealed high binding potency and cell signaling in RC-4B/C cells [132].

### 4.4. SAR of Kisspeptin/Metastin

**Figure 1:**
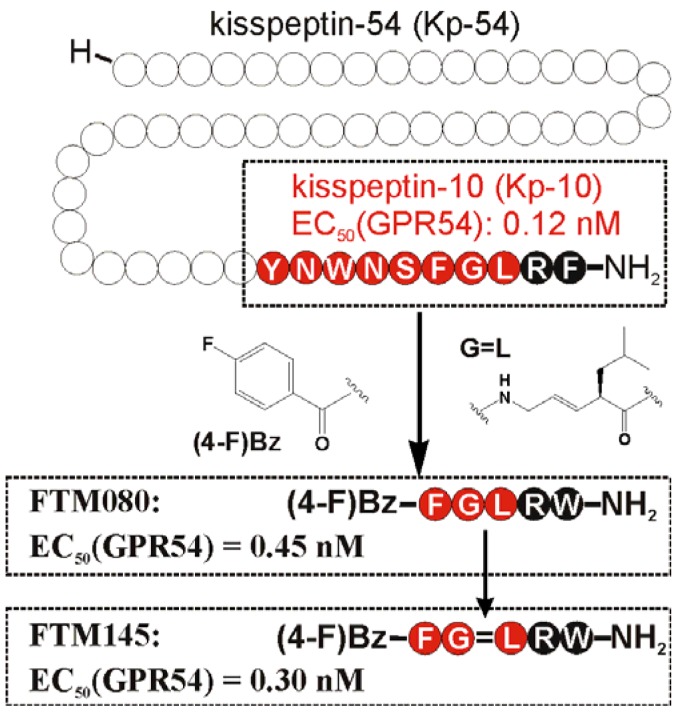
Sequences and bioactivity of down-sized agonists for the kisspeptin receptor (GPR54). Truncated Kp-10 was used as lead structure for developing FTM080 and FTM145 (Scheme is based on Oishi *et al.* [138]). EC_50_ values represent the concentration required for 50% of the full agonistic activity induced by Kp-10 (1 μM) [139].

Since the discovery of the kisspeptin receptor (GPR54) and its full-length ligand Kp-54, numerous peptide and non-peptide ligands have been investigated in structure-activity studies. The smallest highly potent agonist represents the N-terminally truncated Kp-10 and was the lead structure for further structure-activity studies (Figure 1) [15,133]. Later studies revealed that an even minor alteration of the amidated C-terminus is not tolerated at all [134]. Initial *D*-amino acid scanning experiments of Kp-10 revealed that the five C-terminal residues are stereochemically of high importance for proper kisspeptin receptor activation [135]. This is in a line with NMR solution experiments of a Kp-13 in a membrane-like environment (SDS micelles), suggesting a relatively stable, helical conformation from residues 7 to 13 [136]. Similar results were obtained by molecular modeling, showing that Kp-10 exhibits a helical structure of the C-terminal 7 residues in an α- and 3_10_-characteristic [133]. In Ala-scanning experiments of Kp-10, the residues Phe^6^, Arg^9^, and Phe^10^ resulted in a high loss of agonistic activity [133,135,136], which structurally fits well with the structural data, as these three residues lie on one face of the helix and define a pharmacophore site for kisspeptin [136]. Structure-activity studies at the C-terminal Phe resulted in an improved activity by substitution with Trp (Table 8) [136,137].

Based on this knowledge, Niida *et al.* developed the significantly down-sized analogs FM052a and FM053a with Kp-10 like agonistic activity (Table 8) [135]. Extensive optimization of this pentapeptide-based C-terminal kisspeptin analogs resulted in the potent kisspeptin receptor (GPR54) agonist: H-Amb-Nal(2)-Gly-Leu-Arg-Trp-NH_2_ (compound 34; Table 8) [134]. Further SAR and QSAR studies, performed on the N-terminal acyl groups of the pentapeptide agonists demonstrated that an aromatic acyl group and the inductive electronegative as well as small substituents at 4-position of the aromatic ring contributed to the agonistic activity. This resulted in the identification of the kisspeptin receptor (GPR54) activating agonist FTM080, which is equipotent with Kp-10 (Figure 1) [140]. The analog FTM145 was developed to prevent degradation by proteinases under physiological conditions, in which the (*E*)-alkene dipeptide isostere at the Gly-Leu site was inserted. FTM145 revealed a higher stability in murine serum and resistance to matrix metalloproteinase mediated cleavage as its half-life was increased up to 38 h, whereas Kp-10 was completely digested after 1 hour [139,141]. Kp-10 exhibited highly potent binding affinity and receptor activation at both NPFF receptors, whereas FTM145 and FTM080 showed reduced bioactivity toward NPFFRs, yet retaining equipotent bioactivity as Kp-10 toward kisspeptin receptor (GPR54). Highly selective agonists are as important as specific receptor antagonists for functional investigations.

It should be noted, that Curtis *et al.* also tested several Kp-10 analogs and describe [*D*-Y]^1^Kp-10 to bind to kisspeptin receptor (GPR54) with lower affinity while exhibiting similar bioactivity *in vitro*. However, peripheral administration of [*D*-Y]^1^Kp-10 increased plasma LH and testosterone *in vivo* more potently than Kp-10 itself in mice. [*D*-Y]^1^Kp-10 was suggested to be more stable for proteolytic degradation compared to endogenous Kp-10. This hypothesis was supported by significantly increased total testosterone levels, measured 60 min after injection of 0.15 nmol [*D*-Y]^1^Kp-10 whereas the same dose of Kp-10 had no significant effect.

## 5. Crosstalk/Therapeutical Potential

**Figure 2:**
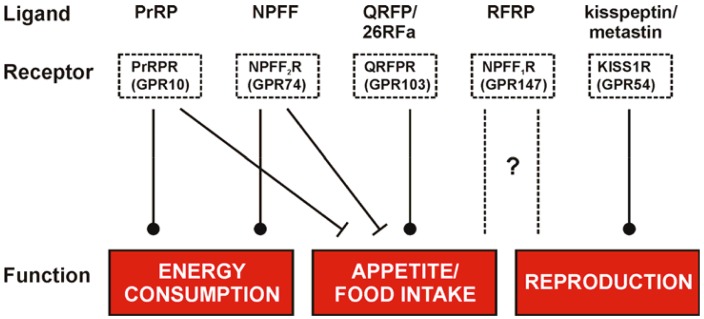
The family of human RFamide peptides and their respective receptors are presented in a summary of our current understanding of their main roles in the regulation of hypothalamic function. The scheme is based on Ebling *et al.* [143]. Details have been reported by Bechtold *et al.* [51] and Osugi *et al.* [144].

The RFamide peptides represent a family with a strong therapeutic potential as they are involved in numerous regulatory mechanisms related to energy homeostasis, reproduction, pain and behavioral processes like food intake, locomotion and stress response. Their roles in the regulation of hypothalamic functions are summarized in Figure 2 and reflect the multiple mediated effects but more importantly their different impact on these functions. Due to the structural homology of the RFamide peptides they are susceptible to address other GPCRs within this family and evoke a so called crosstalk. This fact is a hurdle in investigations of the distinct roles for single RFamide peptides, e.g. the Kp-10 was described to target the NPFF receptors with low nanomolar affinities [138] and PrRP effects were found to be mediated through the NPFF receptors as well [76]. But also the NPFF receptors themselves share high affinities to their endogenous ligands, NPFF and RFRP-3 (NPVF) [28] and the NPFF_2_R has even been described to bind the Y_1_ receptor antagonist BIBP3226. The capability to perform crosstalk is reasonably high for the family of RFamide peptides and thus, selective agonists as well as antagonists are needed to explore the distinct and yet not fully understood mechanism of the evoked diverse pharmacological effects. The development of small ligands might finally lead to small, low molecular weight and lipophilic compounds/ligands as drugs/tools to use them in therapy. As all RFamide peptides take part in fundamental neuroendocrine, behavioral and sensory functions the exploration of selective agonists and antagonists is a fundamental step to gain knowledge of distinct interactions.

### 5.1. Antagonists for the RFamide System

Antagonists, especially highly selective ones are a tool of choice for the investigation of distinct physiological effects mediated by single receptors and their ligands *in vivo.* First steps were made in the development of kisspeptin antagonists, which might provide a valuable instrument to elucidate the physiological and pathophysiological role of kisspeptin in the regulation of reproduction and could offer a unique therapeutic agent for treating hormone-dependent disorders of reproduction, including precocious puberty, endometriosis, and metastatic prostate cancer. The most advanced group of antagonistic compounds was developed for the NPFF receptor ligand system, which represents a promising target for drugs with its impact on nociception, food intake and energy expenditure. They may help to optimize the impact of opiates or improve feeding behavior. Up to now, no specific antagonists have been described for the QRFP receptor (GPR103) and the PrRP receptor (GPR10). But it has to be mentioned, that NPY dosed in the micromolar range showed some antagonistic effects for PrRP [145].

### 5.2. Development of Antagonists for the NPFFR/GnIH System

Several putative antagonists have been reported to target NPFF receptors, e.g. compounds derived from endogenous NPFF ligands. Development of antagonists for the NPFFR/GnIH system included N-terminally truncated peptides, like desaminotyrosyl-FLFQRFamide, dansyl-PQRamide and PFR(Tic)amide [42,146-149]. Unfortunately, most of them act as partial agonists with a low affinity and thus limit their use as pharmacological tools. In 2002, Mollereau *et al.* showed that BIBP3226, a prototypical NPY_1_R non-peptidic antagonist [150], displays high affinities for NPFF receptors [120]. Since then, several studies demonstrated the antagonistic properties of BIBP3226 at NPFF receptors *in vitro* and *in vivo* (Table 9). Nevertheless, its application is limited due to its simultaneous blockade NPY_1_ receptors [64,120,151-153]. It has been suggested that BIBP3226 and related compounds may mediate some of their *in vivo* effects through NPFF receptors rather than through NPY_1_ receptors [154,155]. In 2002, similar compounds to BIBP3226 were described in a patent [156]. Recently, a derivative of the dipeptide RFamide (RF9) has been suggested to be a potent and selective antagonist [157]. Selective binding of RF9 to recombinantly expressed receptors in CHO and COS-1 cells was shown (K_i_-values of 58 nM and 75 nM for NPFF_1_R and NPFF_2_R, respectively) (Table 9). RF9 antagonizes the NPFF-/NPVF-induced agonism in functional assays *in vitro* and i.c.v. and eliminates NPFF-induced pressure and tachycardiac responses. When RF9 was coinjected with NPFF, it was able to act as an antagonist to block delayed and long-lasting heroin-induced hyperalgesia [157]. Further on, recent data demonstrate that RF9 prevents NPFF-induced drops of the body temperature and morphine analgesia in mice [158]. Taken together it is now clear, that NPFF receptors mediate the hypothermia and anti-morphine action of NPFF. Additionally, data revealed a disinhibitory role of NPFF and NPVF in the hypothalamic PVN. The reduction of evoked bicuculline-sensitive inhibitory postsynaptic currents was described, which was blocked by RF9 [159]. However, in an IP accumulation assay, we observed agonistic activity with full efficacy at both NPFF receptors of transiently transfected COS7 cells after stimulation with RF9 (unpublished data). As former studies of RF9 were tested in cAMP-assays or investigated its activity via stimulation of [^35^S]GTPγS binding to hNPFF_2_R membranes, it can be speculated that different signaling pathways of the NPFF receptors are responsible for the observed different effects. Recent reports suggested that the different activation of NPFF receptors could generate the diversity of pharmacological effects *in vivo* [62,160].

It should be noted that a couple of patents have described small nonpeptidergic ligands for NPFF_1_R and NPFF_2_R. In 2003, a patent reported on small templates of quinolino and quinazolino guanidines where different substitutions on the ring system were carried out [161]. In 2004, derivatives of a thiazole guanidine template with different substitutions have been described as NPFF_1_R antagonists, with IC_50_-values in low nM range [162]. Unfortunately, functional data for hNPFF_1_R or hNPFF_2_R as well as binding studies on hNPFF_2_R are missing. A later patent refers to the guanine derivatives linked to an aromatic heterocycle to be used as NPFF receptor antagonists [163]. The most recent patent is based on reference [162], describing further potent and specific, low-molecular antagonists of neuropeptide FF1 receptors with non-peptide or non-peptoid structures [164].

### 5.3. Kisspeptin/Metastin System

Roseweir *et al.* created some potent peptide antagonists for the kisspeptin receptor (GPR54), based on the substitution of Leu^8^ in Kp-10 with *D*-Trp in combination with Ser^5^ substitution by Gly (Table 9) [165]. The selected antagonist peptide 234 (p234) reduced pulsatile GnRH secretion in female pubertal monkeys and was able to inhibit the firing of GnRH neurons in the brain of the mouse. The inhibitory effect of LH release in rats and mice and the blocking of LH rise in postcastrated sheep, rats, and mice indicate that kisspeptin neurons mediate the negative feedback effect of sex steroids on gonadotropin secretion in mammals [165]. Pineda *et al.* extended and refined the *in vitro* and *in vivo* testing of the first kisspeptin antagonist p234 by its continuous infusion, and additionally tagged the leading antagonist compound p234 with an N-terminal penetration sequence (Table 9) [166]. This is predicted to have a higher permeability through the blood-brain barrier and paves the way for new strategy development for systemic antagonism of (or at least part of) the biological actions of endogenous kisspeptins.

More recently, small molecule kisspeptin receptor (GPR54) antagonists with a 2-acylamino-4,6-diphenylpyridine scaffold have been reported (Table 9) [167,168]. Kobayashi *et al.* used a combinatorial chemistry technology to identify a 2-furoyl group to be the most suitable 2-acyl group of all tested 2-acylamino-4,6-diphenylpyridine derivatives, and finally these structure-activity relationship studies led to compound 9l with an IC_50_ value of 3.7 nM in a kisspeptin receptor (GPR54) binding assay. Moreover, these compounds showed apparent antagonistic activity in a cellular functional assay [167]. The optimized compound 15a exhibited high affinity to human and rat kisspeptin receptor (GPR54), apparent antagonistic activity, and high brain exposure. In addition, intravenous administration of 15a to castrated male rat suppressed the plasma LH level, which indicates the possibility of a small molecule kisspeptin receptor (GPR54) antagonist as a novel drug for sex-hormone dependent diseases [168].

### 5.4. Development of Selective NPFF Receptor Agonists

Gaubert *et al.* have reported the discovery and characterization of the first nonpeptidic selective NPFF_2_R agonists. They demonstrated that systemic administration of the selective NPFF_2_R agonists (compound 1 and 3; Table 10) were active in various pain models *in vivo*, whereas administration of a nonselective NPFF_1_R and NPFF_2_R agonist (compound 9; Table 10) increases sensitivity to noxious and non-noxious stimuli [123].

By performing two functional assays (R-SAT and cAMP assays) in addition to binding assays using ^125^I-NPFF, Lameh *et al.* investigated the activity profiles of several novel nonpeptidic, small molecule NPFF agonists for recombinant human NPFF_1_ and NPFF_2_ receptors (Table 10). In this study, they could identify compounds that, according to the performed *in vitro* functional assays, are selective agonists for NPFF_2_R (*i.e.*, AC-263093) and nonselective agonists (AC-262616) for NPFF receptors (Table 10). Additionally they found AC-262620 and AC-262970 to be selective NPFF_1_R antagonists, which bind NPFF_1_R with low nanomolar affinity but behaved as full agonists at NPFF_2_R [169]. Using these compounds they were able to clarify the pharmacology of NPFF receptors after systemic administration. Further on, the *in vivo* results provide evidence for the divergent roles of NPFF receptor subtypes in the modulation of nociception. They could demonstrate that NPFF_1_R activation is pronociceptive, whereas NPFF_2_R activation is antinociceptive [169].

## 6. Concluding Remarks

In recent years, the essential role of RFamide peptides became more and more clear as the number of physiological functions and effects, the peptides participate in, were growing. The regulatory pathways of the RFamide peptides are involved in prominent nodal points like the cardiovascular system, feeding behavior, locomotion, nociception, energy homeostasis reproduction or cancer metastasis. On the one hand, perturbation within these signaling pathways might lead to serious syndromes but on the other hand the RFamide family opens the field to modulate a plethora of undesired effects in a positive manner. This issue is becoming increasingly important, because cancer, eating disorders, failed reproduction or the cardiovascular diseases are problems of our modern society and an ascending role will be their treatment by drugs. To address the correct signaling pathway in a desired manner it is indispensable to further elucidate the role of the distinct RFamide peptides. Therefore, highly selective agonistic and antagonistic compounds/peptides are essential and still needed, as the crosstalks within this family, but also to the NPY system are quite complex and not fully understood. First steps were made for NPFF and kisspeptins, but this path has to be followed further to benefit from the huge modulatory potential of the RFamide peptide system. Herein, we summarize the diverse physiological roles of the RFamide peptide family and provide detailed insight into the latest structure-activity and structure-affinity relationship studies.

## Figures and Tables

**Table 1:** Comparison of amino acid sequences of endogenous RFamide peptides in human.

**Group**	**Peptide**	**Sequence**	**Ref.**
NPFF group	NPFF	SQAFLFQPQRF-NH_2_	[5]
	NPAF	AGEGLNSQFWSLAAPQRF-NH_2_	[5]
GnIH group	RFRP-1 (NPSF)	MPHSFANLPLRF-NH_2_	[6,13]
	RFRP-3 (NPVF)	VPNLPQRF-NH_2_	[6,13]
26RFa group	43RFa (QRFP)	<EDEGSEATGFLPAAGEK-TSGPLGNLAEELNGYSRKKGGFSFRF-NH_2_	[7,14]
	26RFa	TSGPLGNLAEELNGYSRKKGGFSFRF-NH_2_	[7,14]
PrRP group	PrRP31	SRTHR-HSMEIRTPDINPAWYASRGIRPVGRF-NH_2_	[8,9]
	PrRP20	TPDINPAWYASRGIRPVGRF-NH_2_	[8,9]
Kisspeptin group	kisspeptin-54	GTSLSPPPESSGSRQQPGLSAPHSRQI-PAPQGAVLVQREKDLPNYNWNSFGLRF-NH_2_	[10,15]
	kisspeptin-14	DLPNYNWNSFGLRF-NH_2_	[10,15]
	kisspeptin-13	LPNYNWNSFGLRF-NH_2_	[10,15]
	kisspeptin-10	YNWNSFGLRF-NH_2_	[10,15]

Pyroglutamic acid is shown as < E.

**Table 2:** Overview of putative RFamide receptors in human.

**Ligand**	**Protein names**	**Known gene names**	**Ref.**
RFRP-1 (NPSF), RFRP-3 (NPVF)	Neuropeptide FF receptor 1 (NPFF_1_R)	**NPFFR1** GPR147, NPFF1	[13,16]
NPFF, NPAF	Neuropeptide FF receptor 2 (NPFF_2_R)	**NPFFR2** GPR74, NPFF2	[16,17,21]
43RFa (QRFP), 26RFa	Pyroglutamylated RFamide peptide receptor (QRFPR)	**QRFPR** GPR103	[18,19]
Kisspeptins	Kisspeptin receptor	**KISS1R** AXOR12, GPR54	[20,22]
PrRP31, PrRP20	Prolactin-releasing peptide receptor (PrRPR)	**PRLHR** GPR10, GR3	[8]

**Table 3:** Affinities of NPFF-related peptides determined in the dorsal horn of the rat spinal cord [118,119].

**NPFF derived analogs**	**Sequence**	**NPFF receptors a K_i_ [nM]**
NPFF	F-L-F-Q-P-Q-R-F-NH_2_	0.34 ± 0.07
[N-Ac]NPFF	**acetyl**-F-L-F-Q-P-Q-R-F-NH_2_	0.74 ± 0.16
NPFF(4-8)	Q-P-Q-R-F-NH_2_	20.9 ± 3.1
NPFF(5-8)	P-Q-R-F-NH_2_	15.5 ± 2.3
NPFF(6-8)	Q-R-F-NH_2_	300 ± 45
[Tyr^1^; *D*-Pro^5^]NPFF	**Y**-L-F-Q-**[*D*-Pro]**-Q-R-F-NH_2_	30.0 ± 4.0
[Lys^7^]NPFF	F-L-F-Q-P-Q-**Lys**-F-NH_2_	245 ± 90
[*D*-Arg^7^]NPFF	F-L-F-Q-P-Q-**[*D*-Arg]**-F-NH_2_	43.2 ± 12.9
[Ala^7^]NPFF	F-L-F-Q-P-Q-**Ala**-F-NH_2_	2359 ± 617
[Tyr^8^]NPFF	F-L-F-Q-P-Q-R-**Tyr**-NH_2_	34.0 ± 10.2
[Hph^8^]NPFF	F-L-F-Q-P-Q-R-**Hph**-NH_2_	915 ± 146
[Phg^8^]NPFF	F-L-F-Q-P-Q-R-**Phg**-NH_2_	6468 ± 682
[Ala^8^]NPFF	F-L-F-Q-P-Q-R-**Ala**-NH_2_	312 ± 73
[*D*-Arg^7^; *D*-Phe^8^]NPFF	F-L-F-Q-P-Q-**[*D*-Arg]-[*D*-Phe]**-NH_2_	373 ± 127
NPFF-OH	F-L-F-Q-P-Q-R-F-**OH**	5178 ± 2195

a Affinity of NPFF-related peptides, inhibiting [^125^I]1DMe specific binding in the dorsal horn of the rat spinal cord. K_i_ values are from recent reports [118,119].

**Table 4:** Binding constants (K_i_) and functional parameters (EC_50_) of RFamide-related peptides on human NPFF receptors from recent reports [28,120-122].

**Peptide**	**Sequences**	**NPFF1**		**NPFF2**		**Ref.**
		**Binding [nM]**	**EC_50_ [nM]**	**Binding [nM]**	**EC_50_ [nM]**	
Pro-NPFF_A_-derived peptides						
NPFF	FLFQPQRFa	2.82 ± 0.06	236 ± 43/12 ± 6	0.21 ± 0.03	3 ± 3	[28,120]
NPFF(2-8)	LFQPQRFa	4.6	140	3.0	8.5	[121]
NPFF(3-8)	FQPQRFa	13	360/26 ± 5	28	25 ± 3	[28,121]
NPFF(4-8)	QPQRFa	21	516 ± 399	69	273 ± 103	[28,121]
NPFF-OH	FLFQPQRF-OH	> 10000		> 1000	> 1000	[120]
hNPAF	AGEGLNSQFWSLAAPQRFa	13.0 ± 2.0	324 ± 30	0.14 ± 0.01	0.53 ± 0.03	[120]
Pro-NPFF_B_-derived peptides/RFamide related peptides						
hRFRP-3 (NPVF)	VPNLPQRFa	0.59 ± 0.07	12 ± 2	23 ± 2	99 ± 28	[28,120]
hRFRP-3-7	PNLPQRFa	0.6	3	110	> 1000	[121]
hRFRP-3-6	NLPQRFa	2.6	28	230	> 1000	[121]
hRFRP-3-5	LPQRFa	2.1	51	76	> 1000	[121]
hRFRP-3-4	PQRFa	15	1270 ± 73	26	904 ± 462	[28,120,121]
hRFRP-1(NPSF)	MPHSFANLPLRFa	2.7	4.8	3.8	21 ± 4/330	[120,121]
hRFRP-1-5	LPLRFa	2.5	23	16	129 ± 23/790	[120,121]
PLRFa	PLRFa	5.4 ± 0.8	82 ± 2	0.51 ± 0.05	6.5 ± 0.9/62 ± 54	[28,120,122]

Data represent mean ± S.E.M.; *K*_i_ = IC_50_ / [1 + *L* / *K*_d_] in which IC_50_ is the concentration of competitor required to displace 50% of specific binding of the radioligand; NPFF_1_ receptors were labelled with [^125^I]YVPNLPQRFa and NPFF_2_ receptors were labelled with [^125^I]EYWSLAAPQRFa by Mollereau *et al.* [120,122]. Yoshida *et al.* used [^125^I]hRFRP-3-8 and [^125^I]NPFF for Binding studies at NPFF_1_R and NPFF_2_R, respectively [121]. EC_50_ is the concentration of agonist that inhibits 50% of the intracellular cAMP production induced by forskolin [120-122] or that evokes 50% of full agonist induced signaling in an inositol phosphate accumulation assay [28].

**Table 5:** Comparison of potency (EC_50_) and efficacy (E_max_) at the human NPFF_1_ and NPFF_2_ receptors for NPFF analogs containing modifications in the RFamide motif [28].

**Peptide**	**Sequence**	**hNPFF_1_ receptor**		**hNPFF_2_ receptor**	
		**EC_50_ [nM]**	**E_max_ [%]**	**EC_50_ [nM]**	**E_max_ [%]**
NPFF	F-L-F-Q-P-Q-R-F-NH_2_	12 ± 6	100	3 ± 3	100
[Ala^7^]NPFF	F-L-F-Q-P-Q-**A**-F-NH_2_	7610 ± 1250	26 ± 4	1228 ± 296	(60 ± 9)
[Cit^7^]NPFF	F-L-F-Q-P-Q-**Cit**-F-NH_2_	3170 ± 640	66 ± 8	2469 ± 1167	59 ± 6
charged side chains/missing guanidine group					
[Lys^7^]NPFF	F-L-F-Q-P-Q-**K**-F-NH_2_	1290 ± 340	83 ± 14	565 ± 21	(81 ± 9)
[Orn^7^]NPFF	F-L-F-Q-P-Q-**Orn**-F-NH_2_	3510 ± 1000	57 ± 12	1692 ± 237	74 ± 1
arginine side chain alterations					
[MMA^7^]NPFF	F-L-F-Q-P-Q-**N^ω^MeArg**-F-NH_2_	242 ± 7	109 ± 2	44 ± 19	101 ± 24
[ADMA^7^]NPFF	F-L-F-Q-P-Q-**N^ω^N^ω^MeArg**-F-NH_2_	1040 ± 500	75 ± 4	236 ± 38	96 ± 2
[Agb^7^]NPFF	F-L-F-Q-P-Q-**Agb**-F-NH_2_	1390 ± 180	31 ± 2	1219 ± 1017	96 ± 13
[Agp^7^]NPFF	F-L-F-Q-P-Q-**Agp**-F-NH_2_	3200 ± 160	17 ± 2	1524 ± 211	54 ± 3
aliphatic side chains & aromatic side chains					
[Ala^8^]NPFF	F-L-F-Q-P-Q-R-**A**-NH_2_	ND	-	ND	(25 ± 4)
[Nle^8^]NPFF	F-L-F-Q-P-Q-R-**Nle**-NH_2_	659 ± 73	93 ± 11	287 ± 159	(83 ± 5)
[Cha^8^]NPFF	F-L-F-Q-P-Q-R-**Cha**-NH_2_	44 ± 11	91 ± 25	17 ± 8	(98 ± 4)
[Tyr^8^]NPFF	F-L-F-Q-P-Q-R-**Y**-NH_2_	301 ± 116	91 ± 22	70 ± 34	(83 ± 1)
[Trp^8^]NPFF	F-L-F-Q-P-Q-R-**W**-NH_2_	3410 ± 120	55 ± 25	205 ± 112	(99 ± 8)
[His^8^]NPFF	F-L-F-Q-P-Q-R-**H**-NH_2_	2180 ± 560	29 ± 3	2750 ± 949	67 ± 2
phenylalanine side chain alterations					
[pMePhe^8^]NPFF	F-L-F-Q-P-Q-R-**pMePhe**-NH_2_	57 ± 15	83 ± 3	5 ± 1	96 ± 9
[*D*-Phe^8^]NPFF	F-L-F-Q-P-Q-R-**f**-NH_2_	200 ± 51	84 ± 9	132 ± 22	115 ± 5
[Hph^8^]NPFF	F-L-F-Q-P-Q-R-**Hph**-NH_2_	1760 ± 210	61 ± 10	1331 ± 249	98 ± 18
[Phg^8^]NPFF	F-L-F-Q-P-Q-R-**Phg**-NH_2_	1780 ± 514	29 ± 16	1070 ± 481	98 ± 4

ND = EC_50_ value was not determinable as no full receptor activation was observed up to concentration tested (10 μM). E_max_ values were obtained from the IP accumulation assay tested at highest concentration tested (100 μM). E_max_ values in parentheses were estimated at 10 μM. Values are the mean (± SEM) and all data values are from our recent report [28].

**Table 6:** Potencies (EC_50_) of 26RFa and truncated analogs.

**Compound**	**Sequence**	**EC_50_ [nM]**
h43RFa	< EDEGSEATGFLPAAGEKTSGPLGNLAEELNGYSRKKGGFSFRF-NH_2_	7.7 ± 1.5
h26RFa	TSGPLGNLAEELNGYSRKKGGFSFRF-NH_2_	10.4 ± 1.5
26RFa(10-26)	EELNGYSRKKGGFSFRF-NH_2_	37.5 ± 15.2
26RFa(13-26)	NGYSRKKGGFSFRF-NH_2_	95.3 ± 40.7
26RFa(14-26)	GYSRKKGGFSFRF-NH_2_	185 ± 52
26RFa(18-26)	KKGGFSFRF-NH_2_	233 ± 51
26RFa(19-26)	KGGFSFRF-NH_2_	1710 ± 521
26RFa(20-26)	GGFSFRF-NH_2_	739 ± 149
26RFa(21-26)	GFSFRF-NH_2_	NC
[Nva^23^]26RFa(20-26)	GGF-**Nva**-FRF-NH_2_	233 ± 72

NC = not calculable; Pyroglutamic acid is shown as < E; EC_50_-values are presented with S.E.M. All data have been taken from Le Marec *et al.* [126].

**Table 7:** Activities for the PrRP-(19-31)-peptide Analogs.

**PrRP20 analogs**	**C-terminal sequence and numbering**	**Binding K_i_[nM]**	**FLIPR EC_50_[nM]**
PrRP19-31/PrRP20	…-I^25^-R^26^-P^27^-V^28^-G^29^-R^30^-F^31^-NH_2_	5.3	20
PrRP19-31-NHMe	…-I-R-P-V-G-R-F-**NHMe**	4.4	n/a
PrRP19-31-OMe	…-I-R-P-V-G-R-F-**OMe**	35.3	n/a
PrRP19-31-OH	…-I-R-P-V-G-R-F-**OH**	5000	NF
[His(Bzl)^31^]PrRP19-31	…-I-R-P-V-G-R-**His(Bzl)^31^**-NH_2_	4.7	20
[Me_α_Phe^31^]PrRP19-31	…-I-R-P-V-G-R-**Me**_α_**Phe^31^**-NH_2_	215	4950
[Phg^31^]PrRP19-31	…-I-R-P-V-G-R-**Phg^31^**-NH_2_	199	n/a
[Hph^31^]PrRP19-31	…-I-R-P-V-G-R-**Hph^31^**-NH_2_	517	n/a
[*D*-Phe^31^]PrRP19-31	…-I-R-P-V-G-R-***D*-Phe^31^**-NH_2_	887	n/a
[Ala^30^]PrRP19-31	…-I-R-P-V-G-**Ala^30^**-F-NH_2_	NF	NF
[Lys^30^]PrRP19-31	…-I-R-P-V-G-**Lys^30^**-F-NH_2_	NF	NF
[Ala^29^]PrRP19-31	…-I-R-P-V-**Ala^29^**-R-F-NH_2_	94.5	240
[Me_α_Ala^29^]PrRP19-31	…-I-R-P-V-**Me**_α_**Ala^29^**-R-F-NH_2_	375	2270
[*D*-Ala^29^]PrRP19-31	…-I-R-P-V-***D*-Ala^29^**-R-F-NH_2_	5000	NF
[Phg^28^]PrRP19-31	…-I-R-P-**Phg^28^**-G-R-F-NH_2_	4.7	10
[*D*-Val^28^]PrRP19-31	…-I-R-P-***D*-Val^28^**-G-R-F-NH_2_	2640	n/a
[Me_α_Ala^27^]PrRP19-31	…-I-R-**Me**_α_**Ala^27^**-V-G-R-F-NH_2_	63	130
[Ala^27^]PrRP19-31	…-I-R-**Ala^27^**-V-G-R-F-NH_2_	187	n/a
[Lys^26^]PrRP19-31	…-I-**Lys^26^**-P-V-G-R-F-NH_2_	22	120
[Phg^25^]PrRP19-31	…-**Phg^25^**-R-P-V-G-R-F-NH_2_	8.2	10

NF = non-functional; n/a = not available; Eu-(Lys)PrRP31 displacement binding assay of HEK293-ASR1 Cells was performed. All data are from Boyle *et al.* [125]

**Table 8:** Binding parameters (IC_50_) and potencies (EC_50_) of selected kisspeptin analogs.

**Compound**	**Sequence**	**E_max_ [%]**	**IC_50_ [nM]**	**EC_50_ [nM]**	**Ref.**
Kp-10	Y-N-W-N-S-F-G-L-R-F-NH_2_	100.3 ± 7.3 **^a^**	0.12	0.12	[133,138]
NF1	N-**R-N-F**-L-R-F-NH_2_	NT	NT	8000 ± 700	[137]
Trp^7^-NF1	N-**R-N-F**-L-R-**W**-NH_2_	NT	NT	2100 ± 300	[137]
Gly^4^,Trp^7^-NF1	N-**R-N**-G-L-R-**W**-NH_2_	NT	NT	200	[137]
[*D*-Y]^1^Kp-10	[*D*-Y]-N-W-N-S-F-G-L-R-F-NH_2_	NT	3.6 ± 0.3	NT	[142]
FM052a	**BisPy-Amb**-F-G-L-R-**W**-NH_2_	88.9 ± 2.6**^a^**	NT	3.3	[134,135]
FM053a	**Gu-Amb**-F-G-L-R-**W**-NH_2_	93.7 ± 1.8**^a^**	NT	1.4	[134,135]
Compound 34	H-**Amb-Nal(2)**-G-L-R-**W**-NH_2_	88.9 ± 0.4**^a^**	NT	0.82	[134]
FTM080	**(4-F)Bz**-F-G-L-R-**W**-NH_2_	NT	**0.71**	**0.45** ^b^	**[140]**
FTM145	**(4-F)Bz**-F-**G(ψ 1)L**-R-**W**-NH_2_	NT	**0.12**	**0.30** ^b^	**[140]**

Abbreviations: BisPy: bis[(2-pyridinyl)methyl]; Amb: 4-aminomethylbenzoic acid; Gu: guanidine; Nal(2): 3-(2-naphthyl)alanine; ψ 1: (*E*)-CH=CH-; (4-F)Bz: 4-fluorobenzoyl;

a % activity are based on the relative maximum agonistic activity induced by 10 nM of the compounds (%). Maximum agonistic activity signal at 1 μM Kp-10 was used as reference (100%).

b EC_50_ values represent the concentration required for 50% of the full agonistic activity induced by Kp-10 (1 μM). NT = not tested.

**Table 9:** Structures, sequences and binding affinity of selected specific antagonists for the kisspeptin, NPFF_1_ and/or NPFF_2_ receptors.

**Selected antagonistic ligands**		**Target receptor**	**Binding K_i_ [nM]**	Ref.
**Compound**	**Sequence/structure**			
BIBP3226	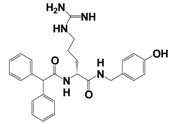	NPFF_1_ (RFRP-3)	16.4 ± 2.3 ^a^	[122]
		NPFF_2_	461 ± 107 ^a^	[157]
RF9	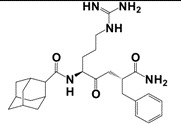	NPFF_1_ (RFRP-3)	58 ± 5 ^a^	
		NPFF_2_	75 ± 9 ^a^	
p210	YNWN**G**FG**w**RF-NH_2_	kisspeptin (metastin)	3 ± NA	[165]
p234	**Ac-a**NWN**G**FG**w**RF-NH_2_	kisspeptin (metastin)	7 ± NA (77.4 ± NA)	[166]
P234-penetratin	**RRMKWKKYa**NWN**G**FG**w**RF-	kisspeptin (metastin)	73.1 ± NA	[166]
9l	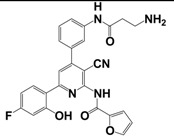	kisspeptin (metastin)	3.7	[167]
15a	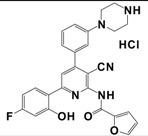	kisspeptin (metastin)	3.6	[168]

a Values are mean ± SEM from three or more separate experiments performed in duplicate. *K*_i_ values were determined by using [^125^I]Tyr-NPFF for hNPFF_2_R and [^125^I]YVP for hNPFF_1_R. NA = value not available; Modifications at the endogenous kisspeptin (metastin) sequence are presented in bold letters and lowercase letters represent residues in *D*-configuration. 9l: N-{4-[3-(β-Alanylamino)phenyl]-3-cyano-6-(4-fluoro-2-hydroxyphenyl)pyridin-2-yl}furan-2-carboxamide hydrochloride; 15a: N-[3-Cyano-6-(4-fluoro-2-hydroxyphenyl)-4-(3-piperazin-1-ylphenyl)pyridin-2-yl]furan-2-carboxamide hydrochloride.

**Table 10:** Binding affinity and/or potency data of potent selective agonists for the NPFF receptor system compared with the NPFF_2_R selective agonists NPFF/NPAF.

**Ligand**	**Sequence/structure**	**Binding**		**Functional Assay ^a^**				**Ref.**
**Selective agonist/antagonist for NPFF receptor**		**K_i_ [nM]**		**E_max_ [%]**	**pEC_50_**	**E_max_ [%]**	**pEC_50_**	
		**NPFFR_2_**	**NPFFR_1_**	**NPFFR_2_**		**NPFFR_1_**		
NPAF	AGEGLNSQFWSLAAPQRFa	3.2 ± 4.0	9.8 ± 0	105 ± 16 ^a^	8.7 ± 0.6	100 ^a^	6.6 ± 0.4	[169]
NPFF	FLFQPQRFa	0.98 ± 1.1	0.9 ± 0	100 ^a^	6.5 ± 0.3		NT	
1	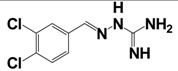	NT	NT	6.0 ± 0.2	68 ± 10	ND	18 ± 7	[123]
3	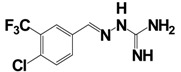	NT	NT	6.3 ± 0.2	114 ± 28	< 5.5	46 ± 0	
9	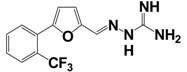	NT	NT	7.0 ± 0.4	73 ±13	7.4 ± 0.2	55 ± 6	
AC-262616		230 ± 11	172 ± 160	73 ± 13 ^a^	7.0 ± 0.4	52 ± 3 ^b^	< 7.4	[169]
AC-263093		1296 ± 942	3320 ± 2890	90 ± 15 ^a^	5.9 ± 0	12 ± 0 ^b^	N.D.	

ND = could not be determined. NT = not tested.

a values measured by R-SAT assays.

b % Eff values reported are those obtained at the highest testable concentration of compound (6 μM), due to toxicity of compounds.
